# Assessing physical activity with the wearable cardioverter defibrillator in patients with newly diagnosed heart failure

**DOI:** 10.3389/fcvm.2023.1176710

**Published:** 2023-05-12

**Authors:** Konstantinos Iliodromitis, Zsuzsanna Balogh, Filippos Triposkiadis, Spyridon Deftereos, Dimitrios Vrachatis, Nana-Yaw Bimpong-Buta, Fabian Schiedat, Harilaos Bogossian

**Affiliations:** ^1^ Clinic for Cardiology and Electrophysiology, Evangelical Hospital Hagen-Haspe, Hagen, Germany; ^2^ School of Medicine, Witten/Herdecke University, Witten, Germany; ^3^ Department of Cardiology, Larissa University General Hospital, Larissa, Greece; ^4^ Medical School, National and Kapodistrian University of Athens, Athens, Greece; ^5^ Clinic for Cardiology, Marienhospital Gelsenkirchen Academic Hospital of the Ruhr University Bochum, Bochum, Germany

**Keywords:** wearable cardioverter defibrillator, life vest, physical activity, ejection fraction, heart failure, sudden cardiac death, remote monitoring

## Abstract

**Background:**

The wearable cardioverter defibrillator (WCD), (LifeVest, ZOLL, Pittsburgh, PA, USA) is a medical device designed for the temporary detection and treatment of malignant ventricular tachyarrhythmias. WCD telemonitoring features enable the evaluation of the physical activity (PhA) of the patients. We sought to assess with the WCD the PhA of patients with newly diagnosed heart failure.

**Methods:**

We collected and analyzed the data of all patients treated with the WCD in our clinic. Patients with newly diagnosed ischemic, or non-ischemic cardiomyopathy and severely reduced ejection fraction, who were treated with the WCD for at least 28 consecutive days and had a compliance of at least 18 h the day were included.

**Results:**

Seventy-seven patients were eligible for analysis. Thirty-seven patients suffered from ischemic and 40 from non-ischemic heart disease. The average days the WCD was carried was 77.3 ± 44.6 days and the mean wearing time was 22.8 ± 2.1 h. The patients showed significantly increased PhA measured by daily steps between the first two and the last two weeks (Mean steps in the first 2 weeks: 4,952.6 ± 3,052.7 vs. mean steps in the last 2 weeks: 6,119.6 ± 3,776.2, *p*-value:  < 0.001). In the end of the surveillance period an increase of the ejection fraction was observed (LVEF-before: 25.8 ± 6.6% vs. LVEF-after: 37.5 ± 10.6%, *p* < 0.001). Improvement of the EF did not correlate with the improvement of PhA.

**Conclusion:**

The WCD provides useful information regarding patient PhA and may be additionally utilized for early heart failure treatment adjustment.

## Introduction

Heart failure with reduced ejection fraction (HFrEF) is a clinical condition associated with increased sudden cardiac death (SCD) risk (1–4). In the early phase of newly diagnosed HFrEF, reversible causes such as ongoing myocardial ischemia, tachyarrhythmias, or acute peri-myocarditis must be treated promptly. Furthermore, despite swift initiation of the evidenced-based medical therapy for heart failure, titration of the of the disease-modifying drugs may be progressively achieved over longer periods (5). During this time frame, the SCD risk may be temporarily high, or cannot be determined. On the other hand, a prophylactic transvenous implantation of a cardioverter-defibrillator (ICD) in patients with severely reduced left ventricular ejection fraction (LVEF) in the early phase after an acute myocardial infarction lacks survival benefit (6, 7).

The wearable cardioverter defibrillator (WCD, LifeVest, ZOLL, Pittsburgh, PA, USA) is a device specifically designed for the temporary detection and treatment of ventricular tachyarrhythmias in patients during a vulnerable period for sudden arrhythmic death. The recently published European Guidelines for the prevention of SCD suggest that the surveillance with the WCD may be prophylactically considered in the early phase after acute myocardial infarction, whereas data on the beneficial effect of the WCD for patients with newly diagnosed non-ischemic cardiomyopathy are sparse (8). The device contains four non-adhesive electrodes positioned orthogonally around the waist (anterior-posterior & right-left), able to produce a two-lead filtered electrocardiogram (ECG) and three self-gelling defibrillation electrodes. This allows an effective and continuous arrhythmia detection from the WCD after combining data from both heart rate and QRS-complex morphology. All detected arrhythmic events are stored in the *LifeVest Network server* (https://lifevestnetwork.zoll.com) and the physician is automatically notified.

Furthermore, WCD has an incorporated accelerometer, which facilitates the counting of the steps, thus providing information about the patients' daily physical activity (PhA). The reliability of the WCD accelerometer as a tool for the assessment of PhA has been already successfully proven compared with the 6-minute-walking test (6MWT) (9).

Registries from Europe and the United States have thoroughly examined the feasibility and safety of the WCD during a vulnerable period for SCD in real world scenarios (10–15). Furthermore, the importance of patient risk stratification over time for SCD after initiation and optimization of heart failure treatment and the reduction of unnecessary ICD implantations has been previously demonstrated (16–18). The VEST-trial examined prospectively a potential benefit of the WCD in patients with reduced LVEF < 35% after AMI (19). The study showed no benefit in this population, however the wearing time with the device was much lower than anticipated (20).

**Figure 1:**
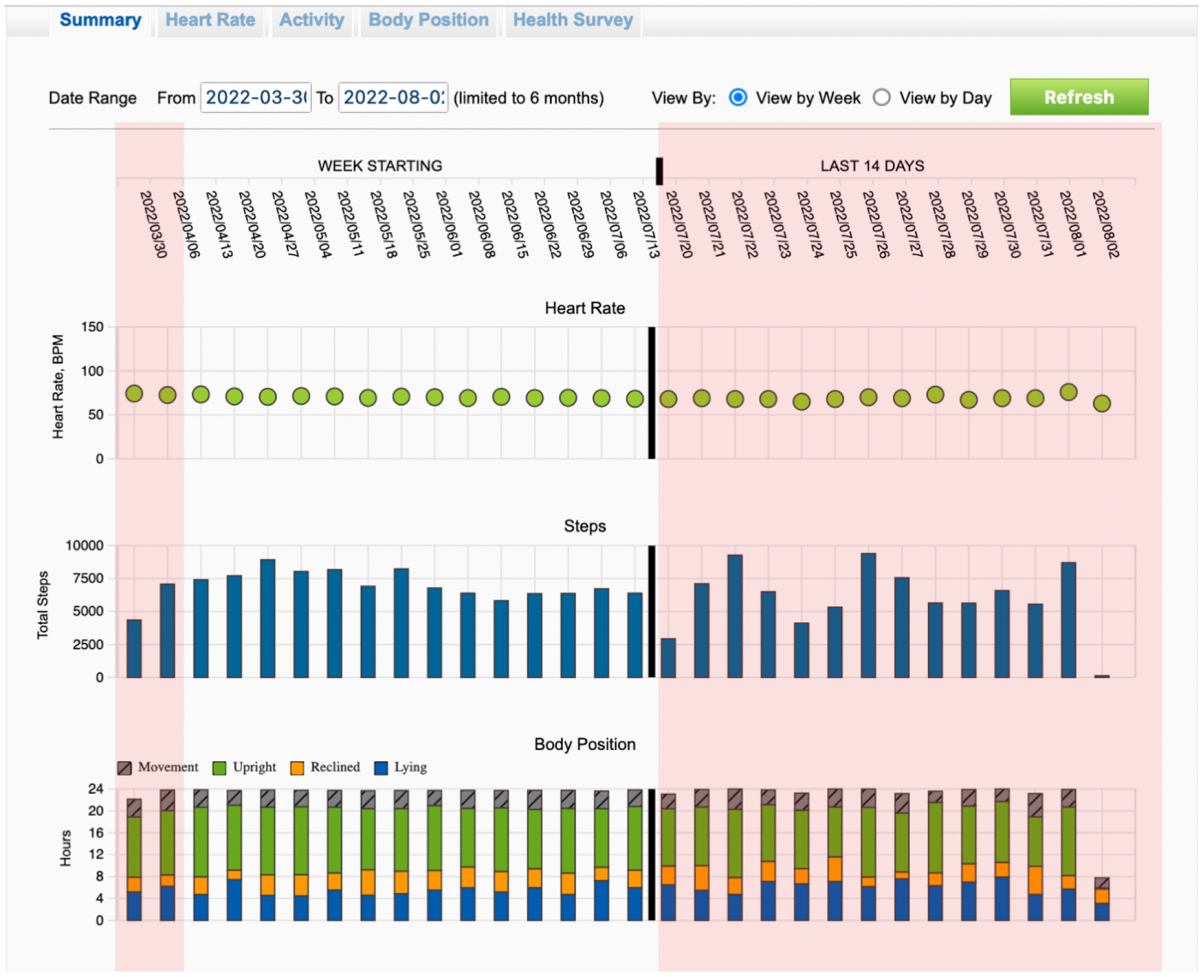
Recordings of the trends from wearable cardioverter defibrillator during the entire surveillance period. Highlighted with red color are the first two weeks and the last 14 days of the total wearing period.

Finally, data selected from the WCD are being stored and can be transmitted to the physician for offline analysis. Available data contain arrhythmic events, heart rate profile and the PhA of the patient in the form of daily steps (Figure 1).

In the present single-center, retrospective study we sought to evaluate the PhA of all patients with newly diagnosed severely reduced LVEF of either ischemic, or non-ischemic etiology, being telemonitored with the WCD until the end of the surveillance period. We also sought to identify clinical factors having an impact to the PhA of the patients.

## Methods

### Study population

**Figure 2:**
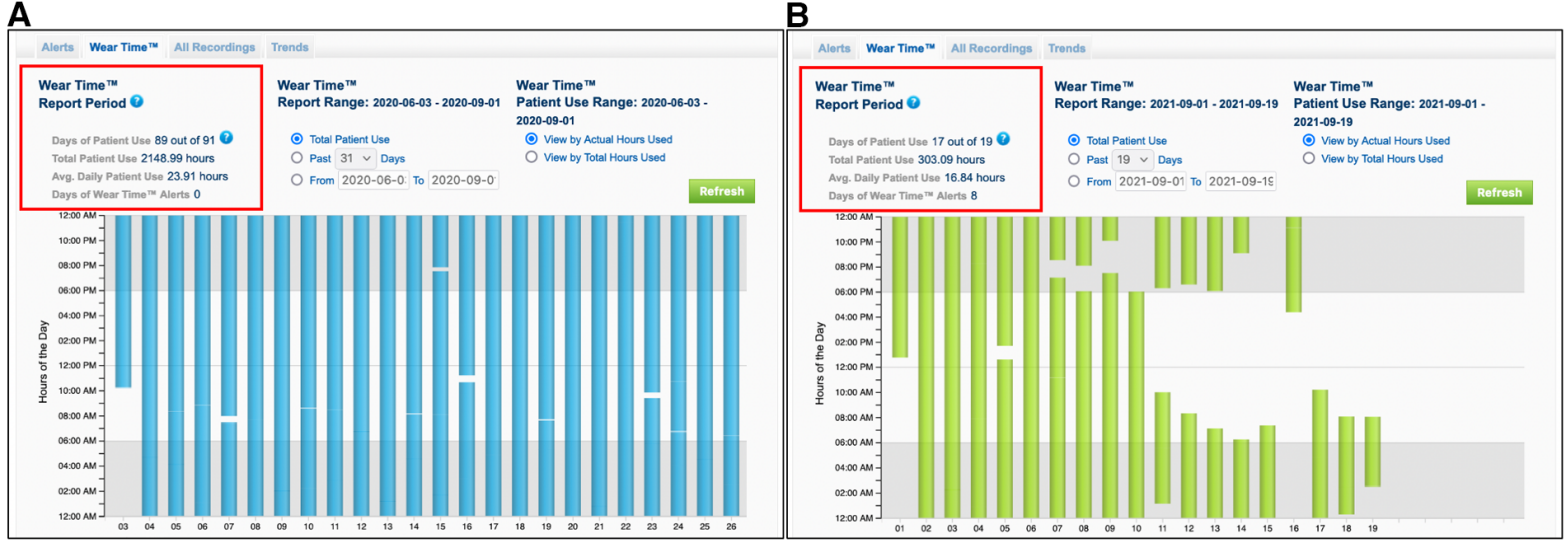
Recordings from the wearing time of the wearable cardioverter defibrillator from two different patients. Patient (**A**) shows a very compliance to the WCD therapy with an average wearing time of 23.91 h per day. Patient (**B**) shows a low compliance to the WCD therapy with an average wearing time of 16.84 h per day, resulting to early termination of the surveillance and exclusion from the study.

A retrospective analysis of all patients treated with the WCD from January 2016 until October 2022 in our clinic was conducted. Inclusion criteria for the study were newly diagnosed non-valvular heart failure, with severely reduced LVEF less than 35% at the day of hospital discharge, of either ischemic, or non-ischemic etiology. Additional inclusion criteria were the duration of the bridging period with the WCD and the compliance to the treatment. Thus, a treatment with the WCD for at least 28 consecutive days and a minimum wearing time of the WCD of at least 18 h daily were prerequisite (Figure 2). Patients with primary electrical heart disease, or being bridged with WCD after removal of their implanted cardioverter defibrillator due to device infection were excluded from the study. The study protocol conformed to the ethical guidelines of the 1975 Declaration of Helsinki.

### Physical activity estimation

All data for analysis were retrieved from the manufacturer database (LifeVest, ZOLL, Pittsburgh, PA, USA). The detection of either ventricular, or supraventricular episodes was noted. The endpoint of PhA was assessed by calculating the average number of daily steps in the first two weeks and comparing it to the average number of daily steps from the last two weeks prior to termination of the surveillance with the WCD. Additionally, we reported the initiation and/or modifications of all guideline recommended heart failure medications affecting the neurohumoral cycle of heart failure at the day of hospital discharge. Finally, we recorded and compared the change of the LVEF of each patient and correlated it with the PhA estimated with the WCD.

### Evaluation of the left ventricular ejection fraction

LVEF evaluation was performed with 2D-transthoracic echocardiography using the modified Simpson's method. Transthoracic echocardiography was performed after reperfusion therapy and/or initiation of medical heart failure treatment (index event) prior to WCD therapy, as well as on scheduled follow-up prior to decision for termination of the WCD therapy.

### Statistical analysis

The SPSS 29 (IBM SPSS Statistics) was used for all statistical analyses of this study. Continuous variables are shown as the mean ± standard deviation (SD). Categorical variables are presented as percentages. Pairwise comparisons of continuous variables were performed using the paired t-test. Factors affecting the results were examined with multivariate linear regression analysis. All of the statistical tests were two-sided at a significance level of 0.05.

## Results

From January 2016 to October 2022 a total of 136 patients had been treated with the WCD. Inclusion criteria were fulfilled in 77 patients, who were included in the analysis. Fifty-five patients were males (70.5%) and the mean age of the study population was 63.7 ± 11.7 years. WCD therapy without further device implantation was terminated in 50 patients (64.9%). In particular, 44 of the patients showed an improvement of their left ventricular function with a LVEF over 35%, whereas six patients denied a permanent device implantation despite no adequate LVEF improvement under heart failure medication at the end of the follow-up. A transvenous one-chamber cardioverter defibrillator (ICD) was implanted in 18 patients (23.4%) and a biventricular cardioverter defibrillator (CRT-D) in 9 patients (11.7%). Episodes of non-sustained ventricular tachycardia were recorded in four individuals (5.2%), all of whom showed no improvement of their LVEF (Table 1).

**Table 1:** Demographic data.

Study population (*n* = 77)			*n*	%
Males			55	71.4
Females			22	28.6
Age (years)			63.7 ± 11.7	
Body mass index (BMI—kg/m²)			28.9 ± 5.7	
Comorbidities			*n*	%
Ischemic Cardiomyopathy			37	48.1
Non-ischemic Cardiomyopathy			40	51.9
Atrial Fibrillation			30	39
	Paroxysmal		7	9.1
	Persistent		14	18.2
	Permanent		9	11.7
Coronary Heart Disease			47	61.4
Chronic obstructive pulmonary disease			15	19.5
Diabetes mellitus			22	28.6
Arterial hypertension			57	74
Overweight (BMI >25 kg/m²)			53	68.8
Arrhythmic events and Outcome			*n*	%
Non sustained ventricular tachycardia			4	5.2
Sustained ventricular tachycardia			0	0
Ventricular fibrillation			0	0
ICD Implantation			18	23.4
CRT-D Implantation			9	11.7
No device implantation			50	64.9
Improvement of LVEF >35%			44	57,1

LVEF, left ventricular ejection fraction; ICD, implantable cardioverter defibrillator; CRT-D, cardiac resynchronization therapy defibrillator.

The average days that our study population carried the WCD was 77.3 ± 44.6 days and the average daily wearing-time of the WCD was 22.8 ± 2.1 h. An improvement of the LVEF was noted at the end of the surveillance period with the WCD (LVEF-before: 25.8 ± 6.6% vs. LVEF-after: 37.5 ± 10.6%, *p* < 0.001). Furthermore, the PhA of the patients increased significantly in the last two weeks of surveillance, compared to the first two weeks (mean steps first two weeks: 4,952.6 ± 3,052.7 vs. mean steps last two weeks: 6,116.6 ± 3,776.2, *p* < 0.001) (Table 2).

**Table 2:** Follow-up Data.

		**First two weeks**	**Last two weeks**	***P*-value**
Average daily steps		4,952.6 ± 3,052.7	6,119.6 ± 3,776.2	<0.001
Average heart rate		73.1 ± 11.1	71.4 ± 10.6	ns
		**Beginning of follow-up**	**End of follow-up**	***P*-value**
Left ventricular ejection fraction (%)		25.8 ± 6.6	37.5 ± 10.7	<0.001
*Δ*-Steps	1,167.1 ± 2,455.9			
*Δ*-LVEF (%)	11.6 ± 10.6			
Wearing Time (hours)	22.8 ± 2.1			
Days carried	77.3 ± 44.6			

*Δ*-Steps, Improvement of physical activity measured in daily steps; *Δ*-LVEF, Improvement of left ventricular ejection fraction.

Multivariate regression analysis was used to evaluate the factors affecting the change of the left ventricular ejection fraction (*Δ*-LVEF). Included factors in the model were the type of cardiomyopathy (ischemic vs. non-ischemic), the wearing time of the WCD in hours, the length of duration the WCD was carried in days and the initiation of each of the guideline recommended heart failure medications (B-Blockers, Angiotensin Converting Enzyme (ACE)-Inhibitors, Angiotensin-1 (AT-1) receptor blockers, Sacubitril/Valsartan, Mineralcorticoid Receptor Antagonists (MRAs) and Sodium-glucose Cotransporter-2 (SGLT2) Inhibitors (Table 3). The only factor that was associated with LVEF improvement was Sacubitril/Valsartan (Table 4).

**Table 3:** Overview of medical treatment for heart failure.

		Prior index event (*n*)	%	After index event (n)	%
B-Blockers		31	40,3	75	97,4
ACE-Inhibitors		18	23,4	19	24,7
AT-1 Receptor Blockers		15	19,5	8	10,4
Sacubitril/Valsartan		4	5,2	50	64,9
MRAs		12	15,6	65	84,4
SGLT2-Inhibitors		5	6,5	34	44,2

ACEs, angiotensin converting enzyme; AT-1, angiotensin-1; MRAs, mineralcorticoid receptor antagonists, SGLT2, sodium-glucose cotransporter-2.

**Table 4:** Factors potentially associated with improvement of left ventricular ejection fraction (*Δ*-LVEF). Results of multivariate regression analysis.

	Unstandardized Coefficients	Standardized Coefficients		t	*P* Value	95.0% Confidence Interval for B	
	B	Std. Error	Beta			Lower Bound	Upper Bound
(Constant)	−34.706	17.534		−1.979	0.052	−69.704	0.292
Type of heart failure	−2.841	2.369	−0.135	−1.199	0.235	−7.570	1.887
Wearing time (hours)	0.964	0.570	0.189	1.693	0.095	−0.173	2.101
Days carried	0.042	0.026	0.177	1.595	0.115	−0.011	0.095
B-Blockers	8.345	7.406	0.126	1.127	0.264	−6.437	23.127
ACEi	12.080	7.121	0.496	1.697	0.094	−2.132	26.293
ARBs	5.404	8.030	0.157	0.673	0.503	−10.623	21.432
Sacubitril/valsartan	15.323	7.132	0.697	2.148	0.035	1.087	29.559
MRAs	1.905	3.296	0.066	0.578	0.565	−4.673	8.484
SGLT-2i	−1.730	2.457	−0.082	−0.704	0.484	−6.634	3.174

Type of heart failure: ischemic vs. nonischemic; ACEi, angiotensin converting enzyme inhibitors; ARBs, angiotensin receptor blockers; MRAs, mineralocorticoid receptor antagonists; SGLT-2i, sodium-glucose cotransporter-2 inhibitors.

Additionally, multivariate regression analysis was used to evaluate the factors affecting the change of the physical activity measured in daily steps (*Δ*-Steps). Included factors in the model were all previously mentioned plus the *Δ*-LVEF. The only factors associated with improvement in physical activity were wearing time of the WCD and the length of duration the WCD was carried (Table 5).

**Table 5:** Factors potentially associated with improvement of physical activity (*Δ*-steps). Results of multivariable regression analysis.

	Unstandardized Coefficients		Standardized Coefficients	t	*P* value	95.0% Confidence Interval for B	
	B	Std. Error	Beta			Lower Bound	Upper Bound
(Constant)	−7,403.588	4,378.612		−1.691	0.096	−16,145.770	1,338.594
Type of heart failure	426.845	581.195	0.087	0.734	0.465	−733.548	1,587.239
Change in LVEF	7.474	29.654	0.032	0.252	0.802	−51.731	66.679
Wearing Time (hours)	300.357	141.208	0.253	2.127	0.037	18.427	582.287
Days carried	13.830	6.518	0.251	2.122	0.038	0.816	26.845
B-Blockers	2,756.052	1,814.530	0.180	1.519	0.134	−866.775	6,378.878
ACEi	−2,094.027	1,765.064	−0.370	−1.186	0.240	−5,618.091	1,430.037
ARBs	−1,349.070	1,955.594	−0.169	−0.690	0.493	−5,253.539	2,555.399
Sacubitril/valsartan	−2,022.073	1,789.791	−0.395	−1.130	0.263	−5,595.506	1,551.360
MRAs	−442.009	801.970	−0.066	−0.551	0.583	−2,043.194	1,159.176
SGLT-2i	50.226	598.536	0.010	0.084	0.933	−1,144.789	1,245.241

Type of heart failure: ischemic vs. nonischemic; LVEF, left ventricular ejection fraction; ACEi, angiotensin converting enzyme inhibitors; ARBs, angiotensin receptor blockers; MRAs, Mineralocorticoid receptor antagonists, SGLT-2i, Sodium-glucose cotransporter-2 inhibitors.

## Discussion

The WCD is a non-invasive option for the treatment of malignant ventricular tachyarrhythmias during a temporary period with increased risk for SCD. Also, the WCD allows daily remote telemonitoring of the patient's PhA during the entire surveillance period.

Currently, the 6*Μ*WT is a well-established and simple medical tool for the evaluation of functional capacity among patients with heart failure (21, 22). Results from Burch AE. et al. showed that the WCD-guided 6MWT provides similar step counts compared to clinician-guided 6MWT, suggesting the reliability and accuracy of step counts with the WCD (9). However, a limitation of the clinical 6MWT remains its applicability in every-day and out-of-hospital settings, as well as its continuity in real life during the entire day and over longer periods. On the contrary, high adherence during the entire day, which is a prerequisite of an effective WCD therapy, enables more accurate and representative assessment of PhA in patients with HFrEF.

The high wearing time compliance with an average daily wearing time of the WCD of 22,8 ± 2.1 h per day was aligned with the average wearing time of previous studies (10–13, 23) assuring a careful daily telemonitoring of the patients. Additionally, the average wearing days that our population carried the WCD was 77.3 ± 44.6 days. Tripp C. et al. examined the PhA with the WCD in a large cohort of patients after acute myocardial infarction (24). Results from that study showed a significant increase of the PhA from the beginning of the prescription of WCD to the end of the therapy. Furthermore, they showed a negative relationship between wearing time over 20 h per day and PhA. Our results confirm their first finding, showing a positive correlation between incremental PhA measured by daily steps and wearing days of the WCD. This may be attributed to a general improvement of health condition. On the contrary, we report a positive correlation between prolonged wearing time and increased PhA. We assume that this may be the result of improved familiarization with the WCD and increased confidence of the patient to exercise after the index event, as none of the administered medical substances were correlated with the improvement of PhA. Similar results have been published by Hillmann et al., examining the PhA with the WCD in a cohort of patients with both ischemic and non-ischemic cardiomyopathy (25), showing a significant increase of the step count between the first and last week of surveillance.

A novel element from the findings of our study is the lack of correlation between the improvement of the LVEF and the PhA of the patients. During the surveillance period with the WCD, a statistically significant improvement of the LVEF was recorded. The analysis of the applied medication showed, a positive correlation between the sacubitril/valsartan initiation and LVEF improvement. None of the remaining prescribed evidenced-based and recommended heart medication did correlate with the improvement of PhA. Moreover, the improvement of the PhA of the patients did not correlate with the improvement of the LVEF. These results are in accordance with previous studies highlighting the limited value of LVEF as a marker for physiological assessment, as this may vary depending on the loading condition of the patient (preload and afterload) and the myocardial contractility (26–28).

**Figure 3:**
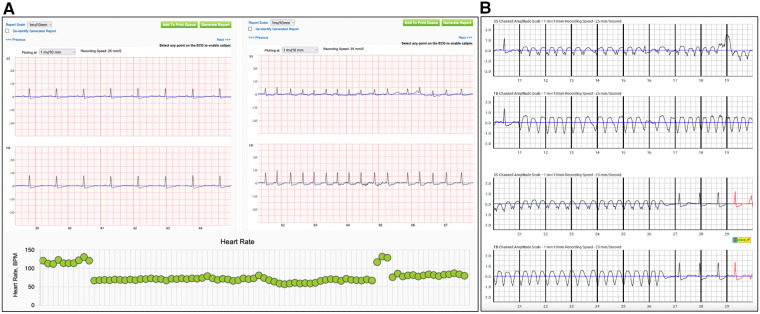
Recordings of alerts from the wearable cardioverter defibrillator during the surveillance period. Patient (**A**) reported worsening of dyspnea, with the electrocardiographic confirmation of atrial fibrillation. In the lower panel are depicted the daily heart rate trends from the same patient, with a sudden increase of the heart rate suggestive for an arrhythmic event. (SS: side-side electrodes, FB: front-back electrodes) Recording from the wearable cardioverter defibrillator of an episode of non-sustained ventricular tachycardia in patient (**B**) with non-ischemic cardiomyopathy. (SS: side-side electrodes, FB: front-back electrodes).

Thus, high adherence to WCD therapy, patient familiarization and education with the device facilitate a high quality daily telemonitoring of PhA. This may lead to early physician interference in cases of patients with good WCD compliance and gradually reduced PhA for the adjustment of the applied medical therapy and avoid unnecessary hospital admissions (Figure 3). None of them showed improvement of their LVEF during the bridging period with the WCD. Although these events may not be enough for conclusions, it highlights the importance of careful interrogation of all available recordings provided from WCD for more accurate, non-invasive risk stratification of the patients.

## Limitations

The retrospective design of the current study remains a limitation. Furthermore, the inclusion criteria for the study population may introduce selection bias in the results, however high compliance to the WCD is prerequisite for effective therapy and the extraction of valid results. Finally, alternative ways for the calculation of PhA, such as steps per hour wearing time, might have been more descriptive.

## Conclusion

The WCD provides useful information regarding the PhA in patients with heart failure, who are having good compliance.

## Data Availability

The original contributions presented in the study are included in the article, further inquiries can be directed to the corresponding author.
